# Imaging tumor and ascites-associated macrophages in a mouse model of metastatic ovarian cancer

**DOI:** 10.1186/s13550-024-01157-8

**Published:** 2024-11-29

**Authors:** Catherine A. Foss, Flonné Wildes, Delia Mezzanzanica, Franca Podo, Chien-Fu Hung, Santosh Yadav, Marie-France Penet Vidaver

**Affiliations:** 1 Department of Radiology and Radiological Science, The Russell H. Morgan, Johns Hopkins School of Medicine, Baltimore, MD USA; 2 Center for Infection and Inflammation Imaging Research, Department of Pediatrics, Johns Hopkins School of Medicine, Baltimore, MD USA; 3 Department of Experimental Oncology, Fondazione IRCCS Istituto Nazionale dei Tumori, Via Amadeo 42, Milano, 20133 Italy; 4 Core Facilities, Istituto Superiore di Sanità, Rome, Italy; 5 Department of Pathology, Johns Hopkins School of Medicine, Baltimore, MD USA; 6 Department of Oncology, Johns Hopkins School of Medicine, Baltimore, MD USA

**Keywords:** Macrophages, iodo-DPA-713, Ovarian cancer, TAMs, Ascites, ID8-Defb29-VEGF

## Abstract

**Background:**

Tumor-Associated Macrophages (TAMs) play a critical role in the pathogenesis and progression of ovarian cancer, a lethal gynecologic malignancy. [^124^I]iodo-DPA-713 is a PET radiotracer that is selectively trapped within reactive macrophages. We have employed this radioligand here as well as a fluorescent analog to image TAMs associated with primary tumors, secondary pulmonary metastases and gastrointestinal tract-associated macrophages, associated with ascites accumulation in a syngeneic mouse model of metastatic ovarian cancer. Intact female C57BL/6 mice were engrafted with ID8-Defb29-VEGF tumor pieces. One month after engraftment, the mice were selected for positive bioluminescence to show primary and secondary tumor burden and were then scanned by PET/MRI with [^124^I]iodo-DPA-713, observing a 24 h uptake time. PET data were overlayed with T_2_-weighted MRI data to facilitate PET uptake tissue identity. Additionally, mice were imaged ex vivo using Near IR Fluorescence (NIRF), capturing the uptake and sequestration of DPA-713-IRDye800CW, a fluorescent analog of the radioligand used here. Additionally, cell culture uptake of DPA-713-IRDye680LT in ID8-DEFb29-VEGF, IOSE hTERT and RAW264.7 cells was conducted to measure tracer uptake in ovarian cancer cells, ovarian epithelial cells and macrophage.

**Results:**

PET/MRI data show an intense ring of radiotracer uptake surrounding primary tumors. PET uptake is also associated with lung metastases, but not healthy lung. Mice displaying ascites also display PET uptake along the gastrointestinal tract while sham-operated mice show minimal gastrointestinal uptake. All mice show specific kidney uptake. Mice imaged by NIRF confirmed TAMs uptake mostly at the rim of primary tumors while 1 mm secondary tumors in the lungs displayed robust, homogeneous uptake of the radio- and fluorescent analog. Ex vivo biodistribution of [^124^I]iodo-DPA-713 showed that contralateral ovaries in middle-stage disease had the highest probe uptake with tissues sampled in mid- and late-stage disease showing increasing uptake.

**Conclusion:**

[^124^I]iodo-DPA-713 and DPA-713-IRDye800CW sensitively identify and locate TAMs in a syngeneic mouse model of metastatic ovarian cancer.

**Supplementary Information:**

The online version contains supplementary material available at 10.1186/s13550-024-01157-8.

## Introduction

Ovarian cancer is a lethal gynecologic malignancy and the fifth leading cause of cancer-related deaths in women in the United States (Cancer Research UK, Statistics). Early detection is a critical unmet need, as patients have significantly improved prognosis when a tumor is discovered at an early stage. Over 70% of epithelial ovarian cancer are diagnosed at advanced stages with metastases in the abdominal cavity and beyond. Disease staging for prognosis and treatment strategy is critical to ensure proper clinical management. Major limitations of radiological evaluation of ovarian cancer arise from the inability to consistently detect small lesions [1]. The availability of multiplexed hybrid imaging systems, such as simultaneous PET/MRI techniques, presents unique opportunities to combine the strengths of anatomic, physiologic and molecular detection within a single exam for the purpose of early detection and staging of ovarian cancer. Studies have shown the potential advantages of using ^18^F FDG PET for ovarian cancer detection and characterization [2].

Tumor associated macrophages (TAMs) play a critical role in ovarian cancer [3]. They represent the most abundant immune population in human ovarian tumor and ascites [4]. The presence of infiltrating TAMs is often correlated with poor patient prognosis. Tissue resident macrophages have been shown to play a role in the invasive progression of metastatic ovarian cancer [5]. Selective depletion of CD163 + Tim4 + macrophages in omentum using genetic and pharmacological tools prevented tumor progression and metastatic spread of disease. TAMs have been shown to facilitate ovarian cancer metastases by supporting tumor spheroid formation by detached cancer cells [6]. TAMs are among several promising targets in host-directed therapies in ovarian cancer due to their high abundance and to their role in facilitating several protumorigenic processes. Moreover, macrophages have been shown to contribute to the immunosuppressive microenvironment observed in ovarian cancer [7].

Non-invasive imaging of macrophages could benefit treatment planning, and response to therapy follow up. Non-invasive techniques for TAM imaging are currently limited. Nanobubbles targeted against CSF1R [8], or FR-beta [9] have been used in preclinical studies for enhanced contrast ultrasound imaging. For MR imaging, Ferumoxytol have been tested using the phagocytic activity of macrophages [10]. The presence of Ferumoxytol creates negative contrast in T2 and T2* weighted images, which can be affected by presence of hemorrhages or calcification, limiting the efficacy of detection of TAMs. [^19^F]-PFC have also been studied in few preclinical studies [11].

PET and SPECT imaging probes have been developed to image macrophages, by targeting surface markers such as CD206 [12], and F4/80 [13]. Translocator protein (TSPO) is an 18-kDa trans-mitochondrial membrane channel involved in cholesterol and other endogenous ligand transport [14]. Though present ubiquitously, TSPO expression is elevated in immunologically pro-tumor activated leukocytes such as microglia and macrophages and has been used as a molecular target for imaging of neuroinflammation. Targeting TSPO for PET imaging of macrophages has been used in preclinical studies, as well as in clinical trials, either with [^18^F]-DPA-714 or [^18^F]-GE-180 [15–17]. The strength of PET lies in its high detection sensitivity and accurate quantification, but PET lacks spatial resolution and tissue contrast. Combining PET with MRI offers great versatility for advanced imaging applications [18]. By merging functional and anatomic information of MRI with molecular PET data originating from a carefully chosen radiotracer, lesion detection can be improved. Few studies using PET-MRI to explore inflammation have been performed, mostly in the context of atherosclerosis. PET imaging of somatostatin receptor using [^68^Ga-DOTATATE and [^18^F]-FET-βAG-TOCA has shown the potential of these probes to image macrophages in large vessel vasculitis and atherosclerosis [19]. In a glioma model, PET-MRI using [^18^F]-fluoroethyltyrosine ([^18^F]-FET) (amino acid metabolism) and *N*,* N*-diethyl-2-[4-(2-^18^ F-fluoroethoxy)phenyl]-5,7-dimethylpyrazolo[1,5-*a*]pyrimidine-3-acetamide ([^18^F]-DPA-714) (translocator protein) was applied to understand the role of glioma-associated microglia and macrophages (GAMMs) in glioma initiation, monitor in vivo therapy-induced GAMM depletion after colony-stimulating factor 1 receptor (CSF1R) targeted therapy, and observe GAMM repopulation after drug withdrawal [20]. The study showed the potential of using [^18^F]-FET and [^18^F]-DPA-714 to image CNS macrophages non-invasively.

In the present study we imaged TAMs in a preclinical model of ovarian cancer. We performed microsurgical orthotopic implantation of the ID8-Defb29-VEGF syngeneic ovarian cancer murine model within the ovary of C57BL/6J mice to image tumor bearing mice at different time points post-surgery to mimic early, intermediate and late-stage disease. To this aim we combined the anatomic imaging capabilities of MRI with the highly sensitive molecular imaging capabilities of PET using [^124^I]iodo-DPA-713, a low molecular weight probe that binds to TSPO at early uptake times and is trapped by reactive macrophages at and beyond 24 h of in vivo uptake, washing out of TSPO-rich healthy tissues before 24 h [21]. While the molecular mechanism for macrophage trapping is not known, final cellular distribution has been proved in multiple models [22–25]. [^125^I]iodo-DPA-713 SPECT and a fluorescent analog (DPA-800) have been already successfully used to specifically image reactive TAMs associated with expanding pancreatic cancer xenografts as well as granulomas associated with *Mycobacterium tuberculosis* infection [21, 26]. Our study was complemented by fluorescent images and histology to better visualize TAMs distribution and confirm the in vivo results.

### Materials and methods

All surgical procedures and animal handling were performed in accordance with protocols approved by the Johns Hopkins University, Institutional Animal Care and Use Committee and Radiation Safety protocols.

#### Murine ovarian cancer model and tumor implantation

ID8-Defb29-VEGF syngeneic tumor model was used in the present study. ID8-Defb29-VEGF derived from ID8, a murine ovarian epithelial papillary serous adenocarcinoma cell line, originating from mouse ovarian surface epithelial cells transformed after multiple passages in vitro [27]. These ID8 cells were modified to constitutively express luciferase and to overexpress VEGF [28]. After intraperitoneal injection, these modified ID8-VEGF cells induce tumor nodules on the visceral and parietal surfaces in the cavity, as well as hemorrhagic ascites [28, 29]. Beta-defensin was then added to the ID8-VEGF cells to increase tumor vascularization [30]. ID8-Defb29-VEGF ovarian cancer cells were grown in RPMI 1640 medium with 10% fetal bovine serum and cultured in standard cell culture incubator conditions at 37 °C in a humidified atmosphere containing 5% CO2.

When injected into the peritoneal cavity they lead to peritoneal spread of tumors and ascites. Here, we performed microsurgical orthotopic implantation of ID8-Defb29-VEGF within the ovary of C57BL/6J mice with a two-step process [31]. We first generated subcutaneous tumors by inoculating a suspension of 2 × 10^6^ ID8-Defb29-VEGF cells in 0.05 ml of Hanks balanced salt solution in the flank of C57BL/6 female mice. Once the subcutaneous tumors reached a size of ∼ 100–200 mm^3^, they were excised, cut into small pieces under sterilized conditions, and implanted surgically onto the ovary of anesthetized C57BL/6 female mice. Sham control mice with similar surgery performed without grafting a tumor piece were used as control.

#### Bioluminescence imaging

Bioluminescence imaging was used to monitor tumor progression, as these cancer cells constitutively express luciferin. Mice were imaged every 2 weeks to assess tumor growth. Experiments were performed when orthotopic tumors reached volumes of ∼ 200–300 mm^3^ (corresponding to a diameter of ∼ 7.5–8.5 mm). A subset of experiments was performed in mice presenting with different disease stages, from early stage when tumors are less than 3 mm with no metastases, to late stage with metastases and ascites present. Bioluminescence imaging was performed using an IVIS Spectrum scanner (Caliper Life Sciences, Hopkinton, MA). To detect the expression of luciferase in vivo, 100 µl of a 30 mg/ml solution of D-luciferin (Promega, P1042, VivoGlo Luciferin, In Vivo Grade, potassium salt) dissolved in PBS was injected intraperitoneally (∼ 3 mg/mouse) 15 min prior to imaging.

#### [^124^I]iodo-DPA-713 radiosynthesis and PET/MRI, PET/CT

[^124^I]iodo-DPA-713 was obtained commercially (3D Imaging, ltd, Little Rock, AK) at ≥ 90% radiochemical purity. Female C57BL/6 mice bearing orthotopic early (*n* = 3), moderate (*n* = 3), disseminated disease (*n* = 3) or a sham surgery (*n* = 3) received intravenous injections of 200 ± 20 µCi (7.4 ± 0.74 MBq) in 100 µL of PBS, pH 7.5. Mice were scanned in pairs after a 24 h uptake using a simultaneous Bruker 7T/30 PET/MRI scanner or a Scintica SuperArgus PET/CT scanner. Mouse pairs were anesthetized using 3% isoflurane in oxygen at 2 L /min and were loaded onto a rat scanner bed observing maintenance 2% isoflurane in oxygen at the same flow rate. Respiration rates were tracked during the experiment using a respiratory pad to adjust the anesthesia level as needed. The PET/MRI images were acquired with three ring Bruker PET insert Si 198. Simultaneous 10 min whole body static PET scan and a T_2_-weighted (T_2_w) 2D coronal scan were acquired for 2 mice placed side by side on the animal bed. 3D maximum likelihood expectation maximization (MLEM) iterative image reconstruction algorithm with a pixel size of 0.5 mm and 16 iterations was used for PET reconstruction. A 72 mm Tx/Rx PET optimized RF coil centered inside the PET detector was used for the MRI acquisition. Multislice T_2_-weighted TurboRARE MRI scan covering the whole body had a FOV of 70 mm x 60 mm, 18 slices, 1 mm slice thickness, TR/TE 3000/32 ms, matrix size of 280 × 240 and 4 averages, resolution 250 μm x 250 μm. In addition, multislice axial scans were acquired using a T_2_-weighted TurboRARE sequence with an FOV of 25 mm x 60 mm, 20 slices, 1 mm slice thickness, TR/TE 2500/25 ms, matrix size of 100 × 240 and 4 averages, resolution 250 μm x 250 μm. After quality assessments, reconstructed PET imaging data were spatially co-registered with MRI data using PV 360 and analysis was performed using PMOD software (Bruker, Boston, MA). PET/CT data were reconstructed using the manufacturer’s software (3D OSEM) and visualized and analyzed using AMIDE (http:/sourceforge.net). 3D MIPs were generated and displayed.

#### DPA-713-IRDye800CW and DPA-713-IRDye680LT ex vivo and in vitro NIRF imaging

Fluorescent probe was synthesized according to well established previous studies [21, 26, 32]. Each mouse (n = one per group) was injected intravenously with 8 nmol of fluorescent tracer in 100 µL of PBS, pH 7.5 followed by a 24 h in-cage uptake. At the time of imaging, mice were sacrificed by anesthetized (isoflurane) cervical dislocation and their body cavity opened and exposed to view. Each mouse was then loaded onto the bed of a LI-COR Pearl Impulse imager. Imaging was performed using a white light (anatomy), 700 nm and 800 nm filter sets. The fluorescent probes were visualized at 700 nm (microscopy study, SI 2–4) and 800 nm (in vivo NIRF) with the 700 nm filter captured tissue autofluorescence. All mouse images were normalized to a chosen exposure time to facilitate comparison.

#### DPA-713-IRDye800CW whole mount tumor NIRF

Primary orthotopic and subcutaneous tumor-bearing mice received 8 nmol of DPA-713-IRDye800CW in 10% DMSO in PBS, pH 7.5 intravenously. The mice were sacrificed 24 h later and the tumors were harvested and promptly frozen on dry ice. The tumors were kept at -80˚C until sectioning on a Leica 550 M cryotome. Twenty µm thick sections were made on charged glass and scanned using a LI-COR Biosciences Odyssey scanner using both 700 and 800 nm emission channels.

#### [^124^I]iodo-DPA-713 ex vivo biodistribution

[^124^I]iodo-DPA-713 was acquired from 3D Imaging Ltd. Each mouse (n = >= 4 mice per group) was injected in the lateral tail vein with 3.7 ± 0.37 MBq (100 ± 10 µCi) of radiotracer in 100 µL of PBS. After 24 h uptake in home cages, mice were euthanized by cervical dislocation and selected tissues were rapidly removed for weighing, followed by counting in a gamma counter (Perkin Elmer Wizard 2480 automatic gamma counter) along with two 10% standards. Uptake values are expressed as percent injected dose per gram of wet tissue.

#### Statistics

Ex vivo biodistribution tissue values in % ID/g were compared using a two-tailed Student’s T-test to determine significance. P values < 0.05 were considered statistically significant. We compared early vs. intermediate, early vs. late, intermediate vs. late, as well as each stage vs. sham controls.

#### Cell lines and Immunofluorescence

Human hTERT-immortalized normal ovarian surface epithelial cells IOSE hTERT 64 were cultured in a medium composed of 199, MCDB 105, 10% fetal bovine serum, 1% glutamine, and G418 (400 ug/mL) [33]. The murine macrophage RAW 264.7 cell line was cultured in DMEM medium with 10% fetal bovine serum. ID8-Defb29-VEGF were cultured in RPMI 1640 medium with 10% fetal bovine serum. All cells were cultured in 8 well chamber slides (Fisher Scientific, 100,000/well) under standard cell culture incubator conditions at 37 °C in a humidified atmosphere containing 5% CO2 and grown to 80% confluency. Cells were probed in vivo with DPA-713-IRDye680LT for 1 h in culture medium. The cells were then rinsed with PBS, pH 7.4 and fixed at room temperature with paraformaldehyde for 20 min., followed by two 5 min washes in PBS, pH 7.4. Cells were then incubated for 1 h at room temperature with a FITC-conjugated anti-CD68 antibody (Abcam ab955) at 1:250 dilution in PBS 10% FBS according to [21]. CD68 is a receptor complex involved in chemotaxis and oxidized LDL scavenging [34]. Cells were then probed with 1:1000 Hoechst 33432 for 1.5 min. and washed twice for 5 min. each with PBS. The walls of the chamber slide were then removed and mounting medium (Dako Faramount aqueous mounting medium, Novus Bio, Centennial, CO) applied along with a coverslip. Slides were viewed using a Nikon 80i epifluorescent microscope and data visualized using the manufacturer’s software, Nikon Elements.

## Results

### PET/MRI, PET/CT and NIRF imaging of TAMs

**Fig. 1:**
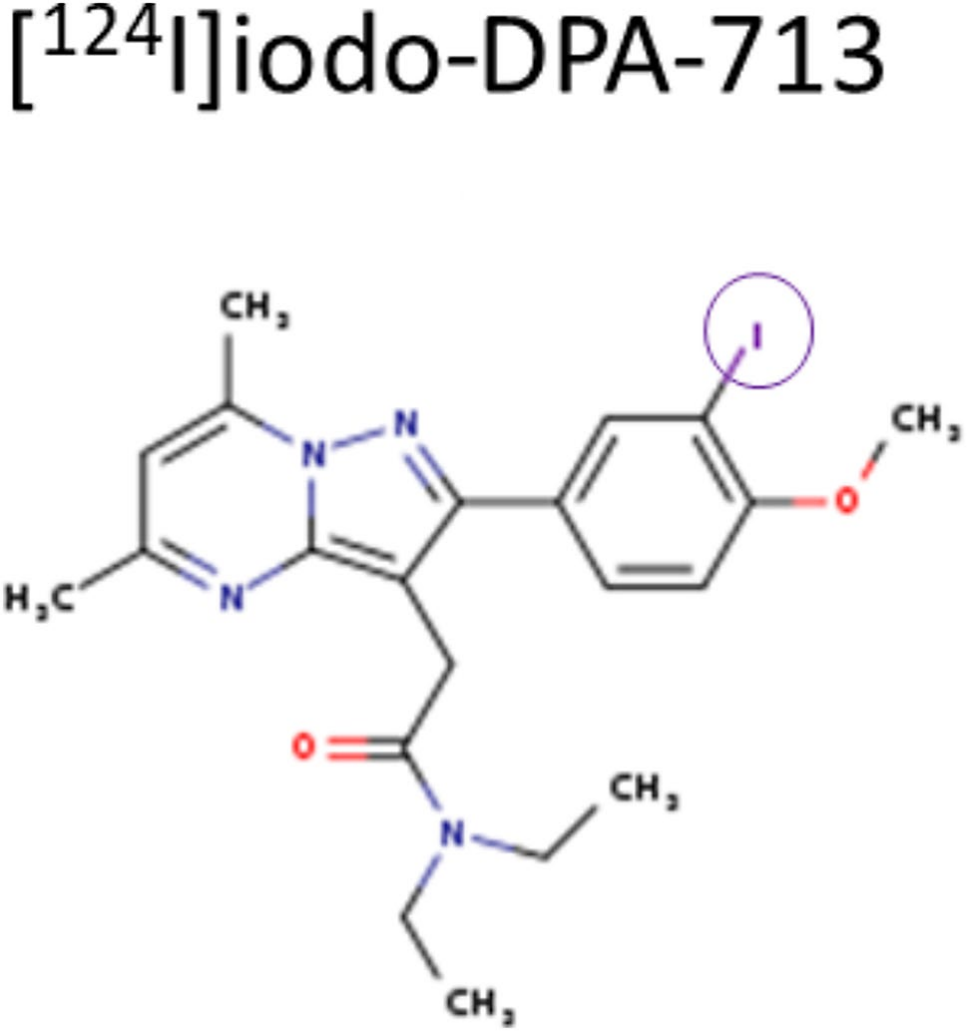
Chemical structures of [^124^I]iodoDPA-713 and DPA-713-IRDye800CW. I-124 radiolabel is circled [21]

**Fig. 2:**
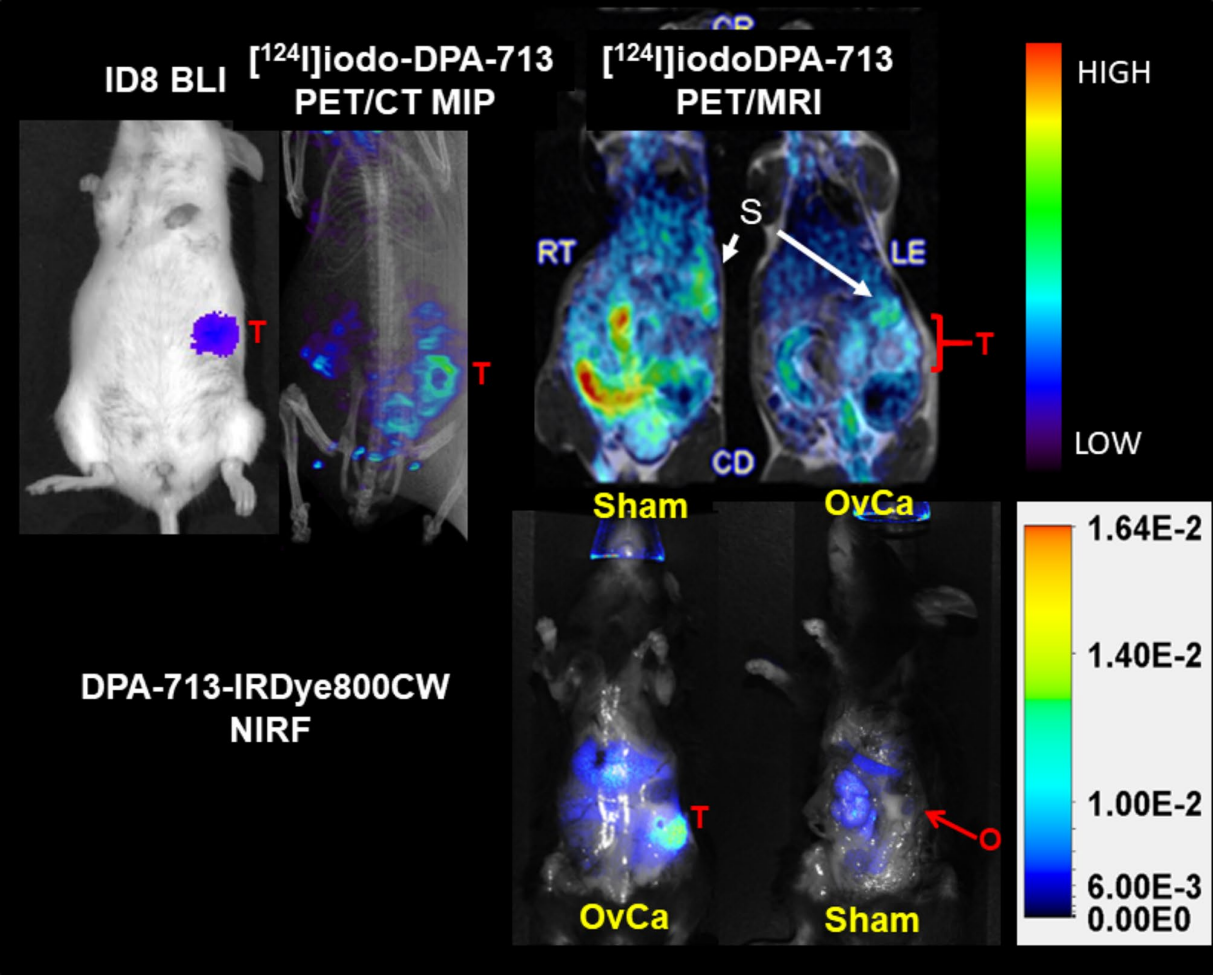
In vivo PET/CT, PET/MRI, BLI and ex vivo imaging of reactive macrophages in orthotopic OvCa and sham operated mice. Mice on upper left in top (BLI and PET/CT) show a mouse with moderate disease and no metastatic spread. Tumor uptake or radiolabeled shown as a MIP reveals a torus-shaped uptake around where the orthotopic tumor resides. The top right two mice (PET/MRI) show radiotracer uptake in sham operated mouse (sham, O) and a mouse with a 1 cm orthotopic tumor (T), showing a ring-spaped uptake of radiotracer around the tumor. [^124^I]iodoDPA uptake in the top panel shows stomach (S, metabolic radioiodine uptake), gastrointestinal tract and uptake by TAMs, which encircle the tumor. The sham surgery shows radiotracer uptake similar to background. The lower panel shows mirror uptake of the fluorescent analog where the tumor (T) is the brightest tissue. The sham mouse again shows background uptake of the fluorescent analog along with gastrointestinal tract and liver uptake

[^124^I]iodo-DPA-713 and DPA-713-IRDye800CW (Radiosyntheses, Fig. 1) were co-administered to orthotopic tumor-bearing mice one day prior to imaging. First, PET/MR and PET/CT images were acquired at 24 h and followed an hour later by ex vivo NIRF or BLI imaging (Fig. 2). In the tumor-bearing mouse in the PET/MR image (Fig. 2 top right), a rim of tracer uptake surrounds the tumor (T, red bracket) while the stomach (S) shows metabolically liberated [^124^I]NaI [35] and the gastrointestinal tract (GI) shows clearance of unbound radiotracer. The sham operated mouse PET/MRI view (Fig. 2 top left) shows metabolic clearance of iodide and unbound radiotracer with a very small amount of uptake at the surgical site, likely related to macrophages involved in wound healing. The PET/CT NIRF alongside BLI clearly shows a ring of uptake where the primary tumor would be. We also acquired images using PET/MRI for superior anatomic colocalization. The NIRF images in the bottom row in Fig. 2 echo the PET uptake where very high fluorescent probe uptake is seen in the tumor (bottom left) with residual unbound tracer exiting through the gastrointestinal tract. The sham-operated mouse (bottom right) shows gastrointestinal clearance with a small amount of fluorescence associated with the wound site over the ovary, indicated with arrow (O).

**Fig. 3:**
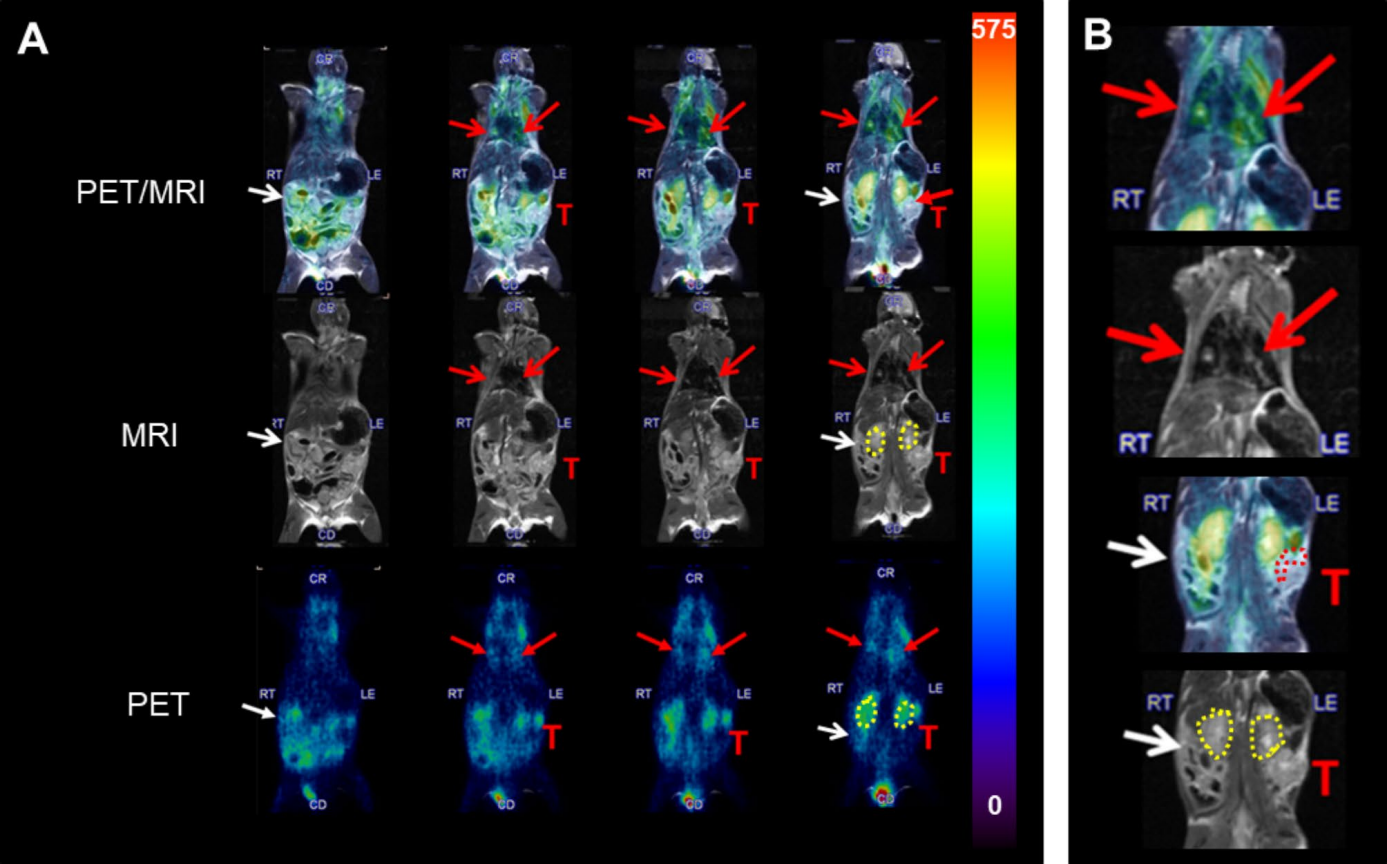
In vivo macrophage imaging in primary and metastatic OvCa. A [^124^I]iodoDPA PET/MRI imaging of a mouse bearing an 8 mm primary orthotopic ID8-Defb29-VEGF tumor (T), peritoneal ascites (white arrows) and several 1 mm pulmonary metastases (red arrows). The primary tumor shows peritumoral uptake of radiotracer in TAMs (dotted lines), even 1 mm metastases with recruited macrophages are easily imaged (B). B Rightmost panel shows magnified views of pulmonary metastases with probe uptake (top) and T_2_ MRI (middle) and a magnified view of primary orthotopic probe uptake by PET with MRI (bottom). Peri-tumoral TAMs uptake in the primary tumor is shown in dotted lines. Scale bar indicates kBq/cc

Mice bearing advanced orthotopic tumors frequently develop ascites. Figure 3 shows PET/MRI data (**A**) of a mouse with a large primary orthotopic tumor (T), pulmonary metastases (red arrows) and ascites (white arrow). The primary tumor has a discontinuous peri-tumoral ring of uptake (dotted red circle in magnification, **B**) while the small metastases show high uptake of radiotracer (**A** and **B**). Gastrointestinal wall adjacent to ascitic fluid also shows high uptake, particularly along the gut wall (**A**, top merged images) but absent in lumen.

### Ex vivo NIRF imaging of TAMs using DPA-713-IRDye800CW

**Fig. 4:**
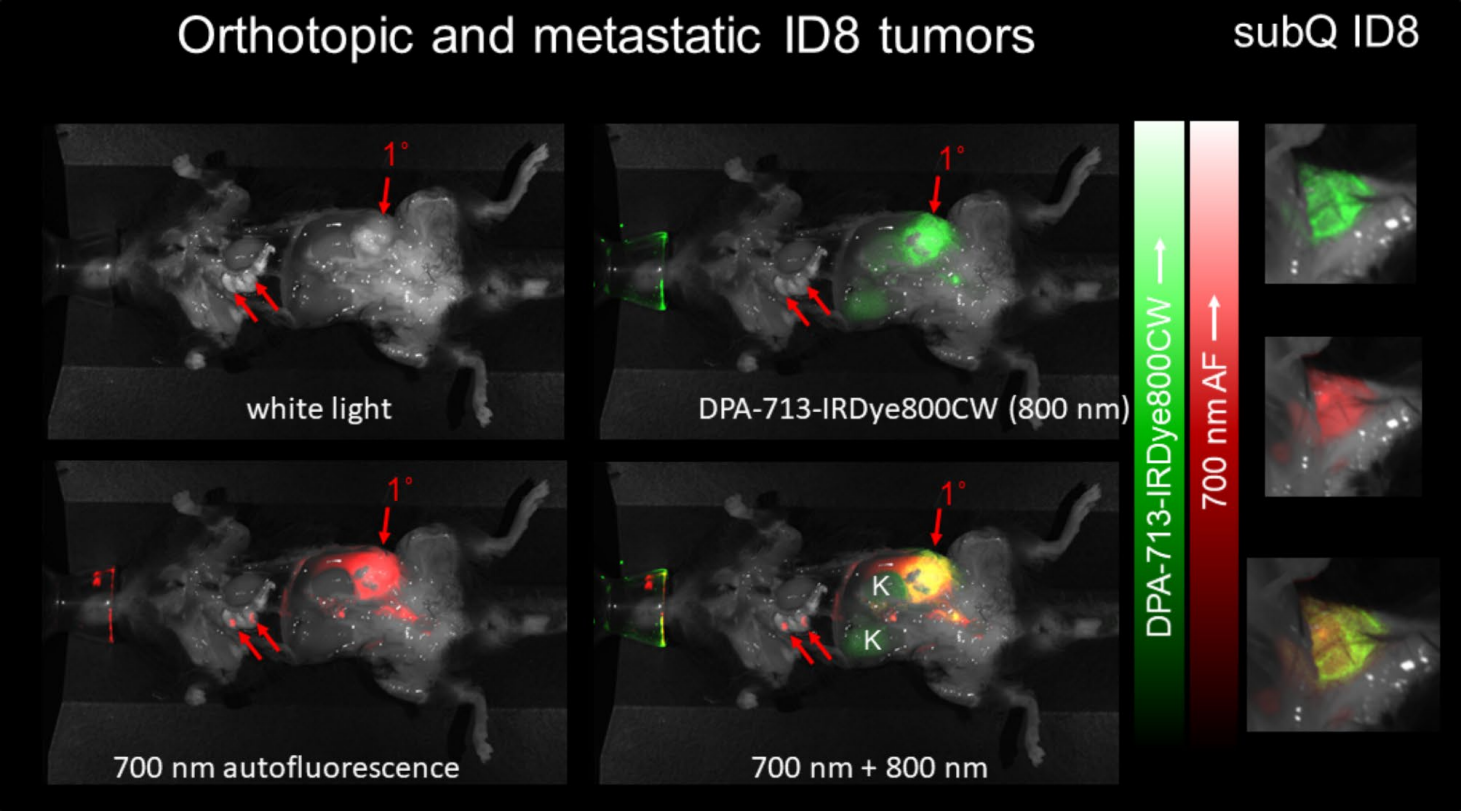
Near IR fluorescence ex vivo imaging of orthotopic, metastatic and subcutaneous ID8-Defb29-VEGF tumors. DPA-713-IRDye800CW (green) exhibits very high whole-tumor uptake in the orthotopic model (1˚), much brighter than the < 1 mm pulmonary metastases (red arrows). The mouse bearing a subcutaneous tumor shows classic peri-tumoral uptake (subcutaneous ID8-Defb29-VEGF in green and in merged 700 + 800 nm channels)

Fig. 4 shows DPA-713-IRDye800CW uptake in primary orthotopic, metastatic and subcutaneous tumors. Very high probe-related green fluorescence is seen in the advanced orthotopic tumor but not the small metastases. The high signal from the primary tumor may be masking comparatively lower signal from the lung metastases. The subcutaneous tumor displays bands of green uptake around the well encapsulated tumor while the red autofluorescent signal is strong in these ID8-def29-VEGF subcutaneous, primary and metastatic tumors. Protoporphyrin IX expression, an endogenous ligand of TSPO [36], has been demonstrated in aggressive ovarian tumors [37] and has been used as a phototherapeutic target in conjunction with 2-aminolevulenic acid [38]. Protoporphyrin IX (PPIX) emits strongly at 700 nm [39] and in Fig. 4 DPA-713-IRDye800CW (green) colocalizes with autofluorescence possibly from endogenous PPIX (red) in orthotopic, metastatic peritumoral subcutaneous tissue and in subcutaneous tumor

### Ex vivo biodistribution of [^124^I]iodoDPA-713 in tumor-bearing mice

**Fig. 5:**
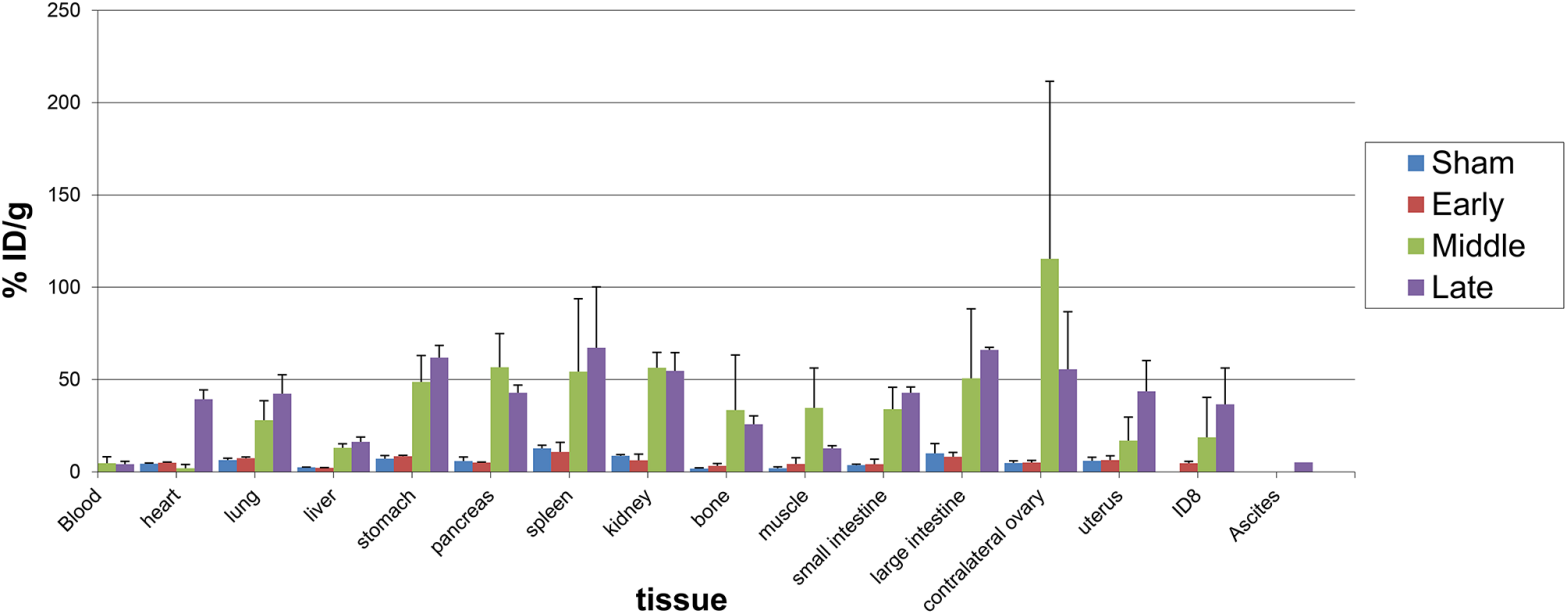
Ex vivo Biodistribution of [^124^I]iodo-DPA-713 in C57Bl/6 mice ± ID8-Defb29-VEGF tumors. Three groups of three to five mice bearing early growth (red bars) orthotopically implanted ID8-Defb29-VEGF, mid growth (green bars) ID8-Defb29-VEGF, late growth (purple bars) ID8-Defb29-VEGF (with ascites) or sham operated were injected with 110 µCi of [^124^I]iodo-DPA-713 followed by a 24 h uptake period. The mice were then euthanized and the indicated tissues were collected. The mice with mid- and late-growth ID8-Defb29-VEGF tumors displayed the highest uptake but not just in the tumors. The mice appear to be systemically inflamed, with elevated uptake across tissues. Contralateral ovary took up a large amount of radiotracer while large intestine was also displaying high uptake, possibly due to inflamed peritoneal macrophages

Female mice were implanted with ID8-defb29-VEGF tumor pieces at different times to allow for a cohort of mice with early, intermediate and late-stage disease to occur at one time along with sham operated mice. Mice were injected with [^124^I]iodoDPA-713 as indicated and observed a 24 h uptake. Sixteen tissues were then harvested and counted as described to define the distribution of radiotracer among the mouse and tissues (Fig. 5). Mice representing intermediate and late-stage disease showed the highest tracer uptake across tissues, possibly reflecting systemic inflammation at those stages. Lung, small and large intestines exhibit increasing uptake correlated with disease stage as expected due to metastases and formation of ascites. There is specific uptake in the ovary due to high levels of TSPO expression [40]. Ex vivo uptake studies in uninfected, tuberculosis (TB) infected and TB + autoblockade mice in ovaries demonstrated that probe uptake ([^125^I]iodo-DPA-713) is specific to ovary [21]. Blockade in BALB/c TB was 80% and blockade in C3HeB/FeJ TB injection was 63%. The uterus and primary tumor also show graded radiotracer uptake corresponding with disease severity. We collected ascitic fluid when it was present but it showed modest radiotracer uptake, suggesting the macrophages that line the GI and peritoneum to be the site of uptake. Statistical evaluation revealed that renal uptake was significantly different across all groups. Early vs. intermediate uptake in kidney was most significant (*P* = 0.0004) while early vs. late was also highly significant (*P* = 0.0009). Most other tissue values had large differences in uptake across groups but standard deviations were also high due to low numbers of mice per group. Unfortunately, no tissue assayed proved a telltale marker between early disease and sham although inflammation went up globally in between early and middle disease, with contralateral ovary providing what could be the best and perhaps specific marker.

### Cell line in vitro uptake of DPA-713-IRDye800CW

**Fig. 6:**
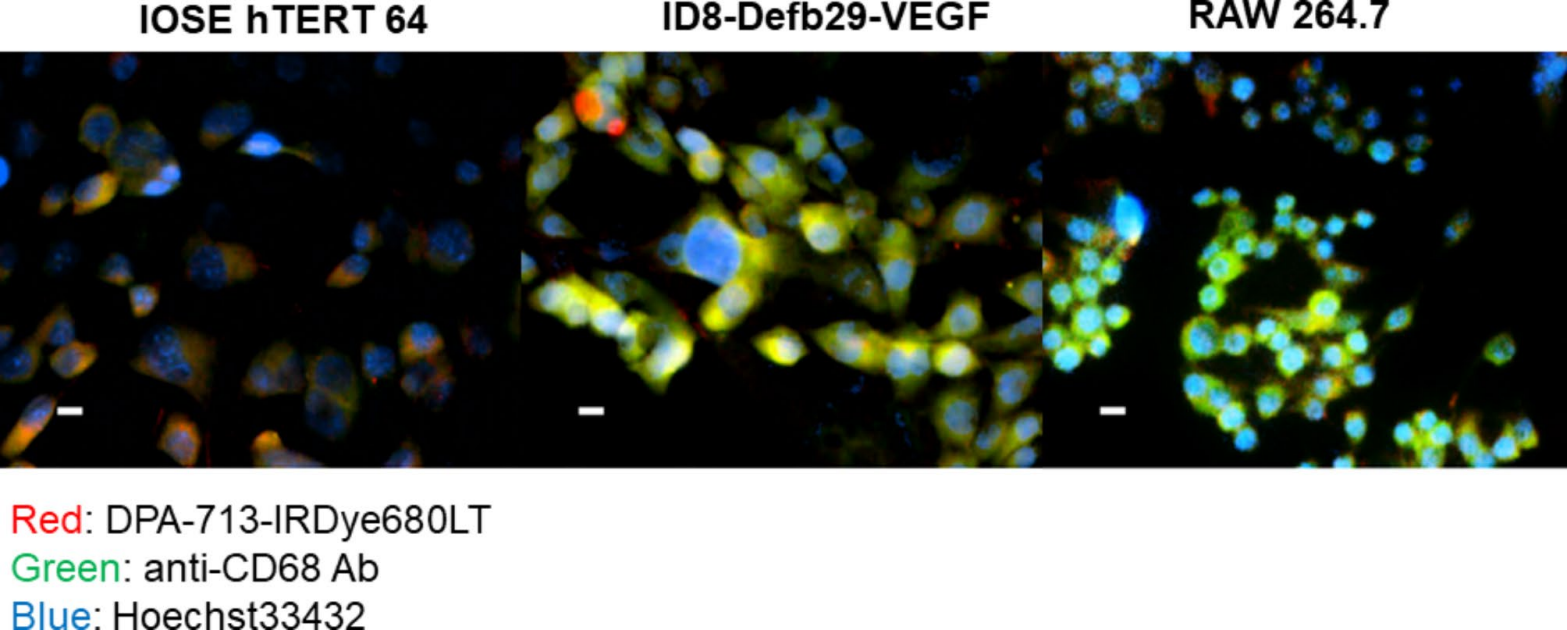
*Live cell uptake of DPA-713-IRDye680LT with subsequent anti-CD68 antibody staining.* All three cell types took up DPA-713-IRDye680LT probe (red) as expected while ID8-Defb29-VEGF carcinoma and RAW264.7 macrophages displayed the highest uptake of DPA probe and CD68 antibody (green)

Three cell lines were incubated as live cell uptake with DPA-713-IRDye800CW. They included immortalized but non-cancerous IOSE hTERT 64, high grade ID8-Defb29-VEGF and murine immortalized macrophage lineage Raw 264.7. Figure 6 shows a composite view of CD68 staining (green), DPA-713-IRDye800CW uptake (red) and Hoechst nuclear staining (blue). Both the ID8-Defb29-VEGF and RAW cells show bright combined channels for CD68 staining and probe uptake (TSPO). The non-cancerous ovarian line (IOSE) shows some TSPO probe binding with very little CD68 staining. CD68 is a receptor complex involved in chemotaxis and oxidized LDL scavenging in monocyte-derived cells and metastatic ovarian cancer [34]. Supporting Information Figs. 2–4 show individual channels for each cell line.

### Estrus cycle and autofluorescence at 700 nm

We collected ovaries and their attached uterine horns in healthy C57BL/6 mice and graded them according to place in the estrus cycle to determine their autofluorescence signal at 700 nm. Figure SI 1 shows a representative set from each stage of estrus at 700 nm (red). While all four stages demonstrate fluorescence in the uteri, only uteri in proestrus shows strong signal, when production of estrogen is high.

## Discussion

We have probed TAMs and ovary proximal to primary orthotopic ID8-Defb29-VEGF tumors, secondary pulmonary ID8-Defb29-VEGF tumors and GI-associated reactive macrophages concomitant with ascites using [^124^I]iodo-DPA-713 and DPA-713-IRDye800CW. Unlike parental [^11^C]DPA-713, which is specific for TSPO, after observing a 24 h uptake time, [^124^I]iodo-DPA-713 is selectively retained by reactive macrophages [21, 41] with washout from background tissues except renal cortex and tissues expressing the sodium iodide symporter, reflecting metabolism of radiotracer and liberation of [^124^I]NaI [35] (salivary gland and stomach). First and second generation TSPO radioligands are all labeled with either C-11 or F-18 and are forced to acquire early timepoints, leaving the lungs, heart and liver bright with TSPO-specific uptake. In the present study, [^124^I]iodo-DPA-713 PET showed uptake aligned with the tumor rim of orthotopic and subcutaneous ID8-Defb29-VEGF tumors and displayed high contrast in secondary 1–2 mm pulmonary metastases. Uptake was also observed in inflamed gastrointestinal tract resulting in ascites, though resolution (0.8 mm for F-18 and 1.2 mm for I-124) is not high enough to precisely localize the uptake to the gut wall. PET signal does localize in the peritoneal cavity with ascitic fluid. Analysis of fluorescent uptake within a non-ascitic mouse showed no GI uptake (green) with some autofluorescent chlorophyll in the gut lumen (red). Quantitated uptake in sham-operated gastrointestinal tract was 5 times lower than for mid-stage and end-stage ovarian carcinoma.

Malignant epithelium has been the primary target for new therapeutics for decades. Only recently and especially with the advent of immune checkpoint therapeutics, have host cell populations been targeted for intervention and diagnostic value [42]. Macrophages, primarily as M2 pro-tumoral phenotype in TAMs [43], play a key role in recruiting vasculature, activating fibroblasts to enable tumor growth, and reducing the immune response of adaptive immune cells [43–46]. Dense concentrations of peritumoral TAMs are associated with poor prognosis in all solid tumors [47–50].

Other cell types are present in the peri-tumoral space as well (NK, lymphocytes, neutrophils, etc.) but in light of the fact that TAMs make up 20–50% of solid tumor volumes, they make an achievable target in terms of sensitivity for PET imaging [51]and allowing physicians to obtain scans for pharmaceutical candidacy and therapeutic efficacy as macrophages are current targets for therapy. A number of folate receptor-α radioligands and others have been used to image ovarian cancer targets (Reviewed here [52]). Most of these probes are antibody-based and require days to achieve high signal-to-noise.

Studies have shown the critical role of TAMs in ID8 tumor progression. The critical role of macrophages in ID8 tumor has been described using a colony-stimulating factor 1 (CSF-1) receptor kinase inhibitor. GW2580 treatment resulted in decreased infiltration of pro-tumorigenic macrophages and in smaller ascites volume. The normalization of the disorganized peritoneal vasculature induced by the treatment showed the importance of TAMs in tumor progression [53]. In a different study, blocking the CXCL12-CXCR4 pathway using AMD3100 in combination with blocking the PD-1-PD-L1 pathway using anti PD-1 antibody prolongs survival of ID8 tumor-bearing mice. The combined therapy prevented the immunosuppressive tumor microenvironment [54].

Tumor infiltrating macrophages (TIMs) are more likely to be tumor suppressive M1 phenotype [55]. The ability to non-invasively image reactive macrophages overlaid onto high resolution anatomy will allow physicians to determine both density of TAMs (tumor rim) and TIMs (tumor parenchyma) in the primary tumor and to determine whether there are observable metastases inside the body where palpation is not an option. Other TAM imaging PET agents have been reviewed in Fernandez B. et al. [48], according to molecular target and M1 vs. M2 activated macrophages. CD206, CD163, arginase, CSF1R, CD68, CCR2 and folate receptor-β as targets with selected specific ligands are summarized. While many of these are specific, the antibody-chelates require days to localize and nearly all end up with high hepatic uptake, obscuring tumors that might be present there and tumors adjacent to the liver in lungs or upper GI.

Malignant ascites build-up occurs more frequently in patients with advanced-stage ovarian cancer. Ascitic fluids are composed of cellular components, including tumor cells, and stromal cells, such as fibroblasts, inflammatory and endothelial cells, and of acellular factors, including cytokines, proteins, and various metabolites [56]. TAMs secrete various soluble factors that induce tumor progression, chemoresistance and immunosuppression. It has been shown that activated gastrointestinal tissue-resident macrophage populations in ovarian cancer ascites can be indicative of patient outcome. Ascites rich in CD163 + macrophages, a M2 phenotype marker, reduced recurrence-free survival [57], while patients with TAMs expressing an interferon signaling signature had a longer overall survival [46]. Targeting TAMs in the tumor microenvironment has been shown to affect ascites accumulation. Targeting their recruitment with GW2580 induced a reduction in ascites volume [53]. Encapsulation of miR-125b, an mRNA within hyaluronic acid nanoparticles, demonstrated repolarization of M2 macrophages to M1 in malignant ascites in an ovarian cancer mouse model, and induced a reduction of ascitic volume [58]. By imaging TAMs non-invasively in the primary tumor and ascitic fluid, treatment planning and treatment efficacy could be more easily assessed.

## Conclusion

We have imaged primary tumoral TAMs with their ovarian tumors, secondary lung metastasis TAMs and activated gastrointestinal tissue-resident macrophages associated with ascites using [^124^I]iodo-DPA-713 PET/MRI and DPA-713-IRDye800CW near IR fluorescence imaging. Tracer uptake is consistent with reported TAMs densities insofar as uptakes were generally surrounding solid primary tumors and were located throughout small secondary tumors that ranged from 1 to 2 mm in diameter. Ascites production, visualized by T_2_-weighted MRI overlayed with PET signal in those observed regions. [^124^I]iodo-DPA-713 PET/MRI is feasible for clinical validation for macrophage-directed therapies and to validate staging for ovarian cancer.

## Electronic supplementary material

Below is the link to the electronic supplementary material.

Supplementary Material 1

Supplementary Material 2

Supplementary Material 3

Supplementary Material 4

Supplementary Material 5

## Data Availability

The datasets used and/or analyzsed during the current study are available from the corresponding author on reasonable request.

## Abbreviations

CSF-1: Colony-Stimulating Factor 1
CSF1R: Colony-Stimulating Factor 1 Receptor
^18^F-DPA-714: N,N-Diethyl-2-[4-(2-^18^F-fluoroethoxy)Phenyl]-5,7-Dimethylpyrazolo[1,5-a]Pyrimidine-3-Acetamide
^18^F-FDG: ^18^F-Fluorodeoxyglucose
^18^F-FET: ^18^F-Fluoroethyltyrosine
GAMMs: Glioma-Associated Microglia and Macrophages
GI: Gastrointestinal Tract
[^124^I]iodo-DPA-713: N,N-diethyl-2-[3-[^124^I]iodo, 4-methoxyphenyl]-5,7-Dimethylpyrazolo[1,5-a]Pyrimidine-3-Acetamide
IR: Infrared
MRI: Magnetic Resonance Imaging
NIRF: Near IR Fluorescence
OvCa: Ovarian Cancer
PBS: Phosphate Buffered Saline
PET: Positron Emission Tomography
TAMs: Tumor Associated Macrophages
TIMs: Tumor Infiltrating Macrophages
TSPO: Translocator Protein
VEGF: Vascular Endothelial Growth Factor
MIP: Maximum intensity Projection
3D OSEM: 3D Ordered-Subsets Expectation Maximization algorithm
