# Supplementary figures and images for: Imaging tumor and ascites-associated macrophages in a mouse model of metastatic ovarian cancer
Source: EJNMMI Res. 2024 Nov 29;14:121. doi: 10.1186/s13550-024-01157-8 (PMC11607259; doi:10.1186/s13550-024-01157-8)

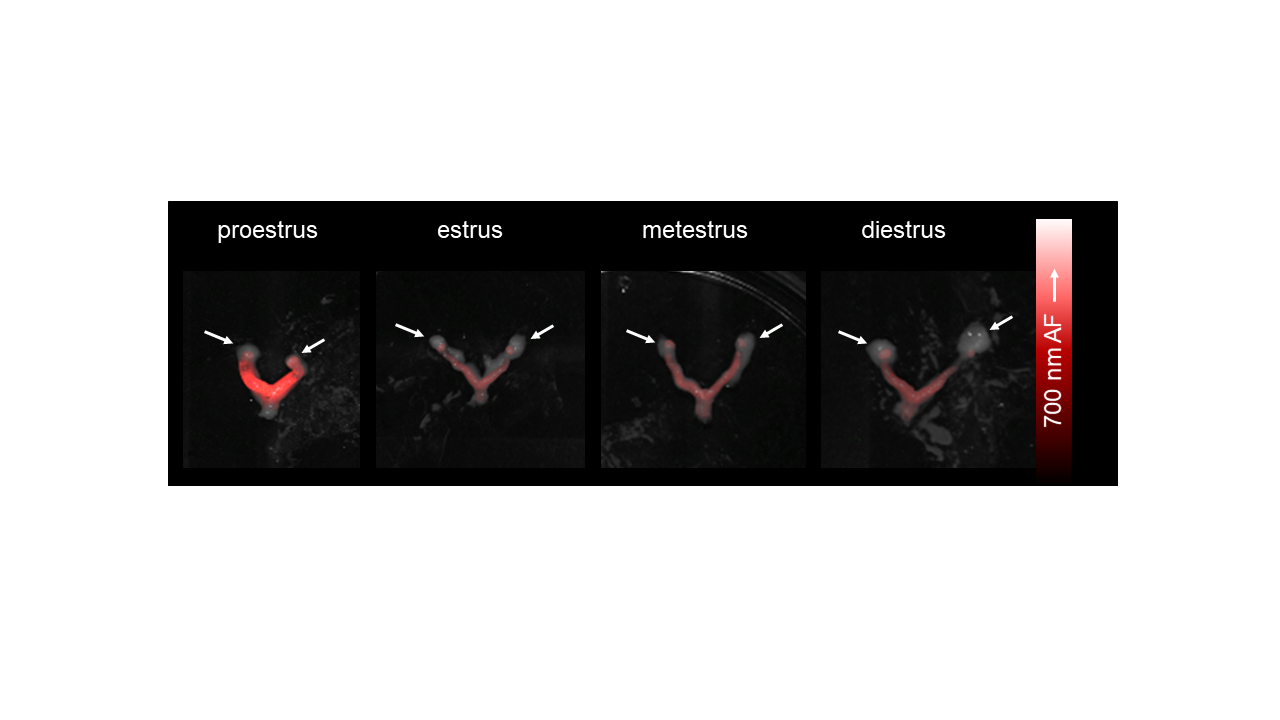

Supplement: Supplementary file 1 — Supplementary Material 1 [file 13550_2024_1157_MOESM1_ESM.tif]

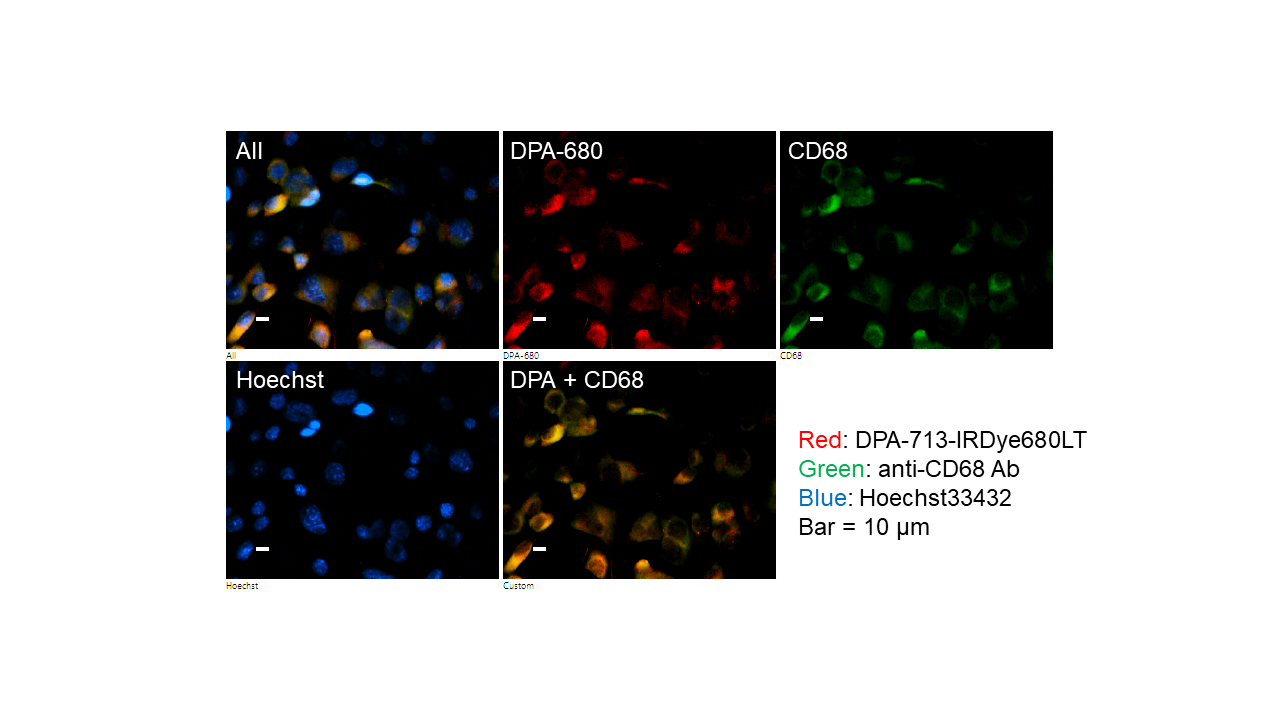

Supplement: Supplementary file 2 — Supplementary Material 2 [file 13550_2024_1157_MOESM2_ESM.tif]

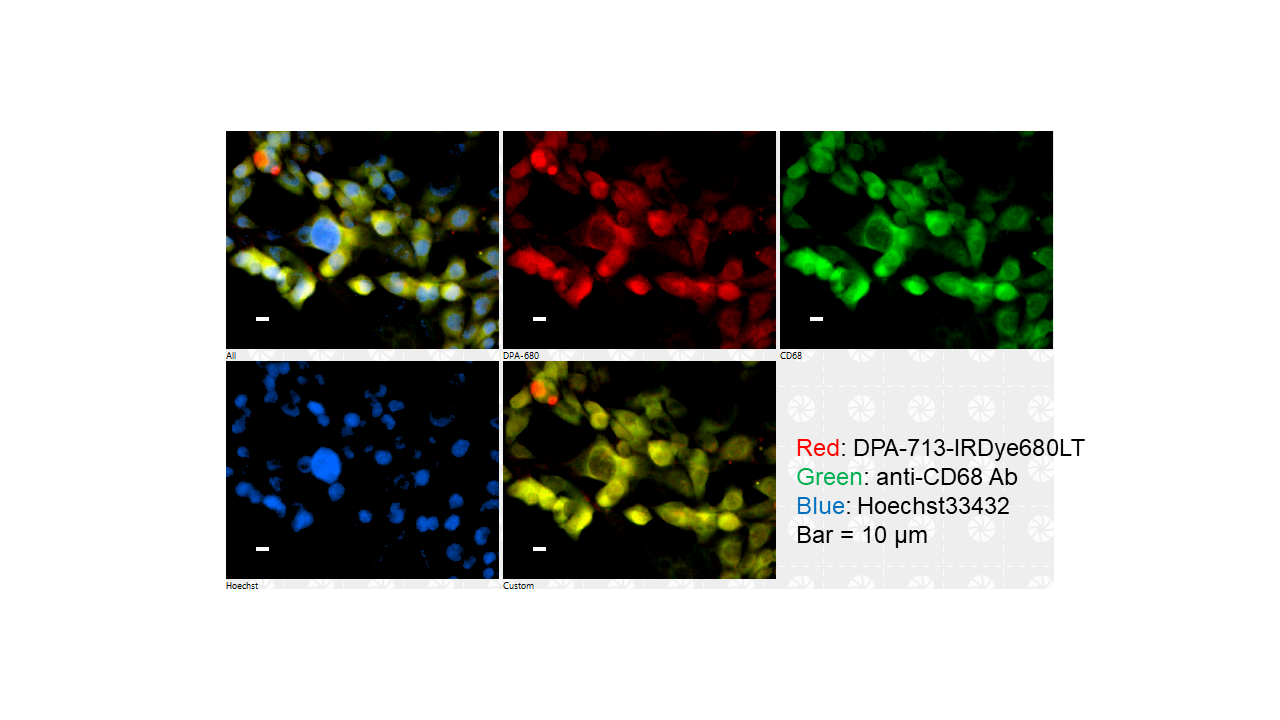

Supplement: Supplementary file 3 — Supplementary Material 3 [file 13550_2024_1157_MOESM3_ESM.tif]

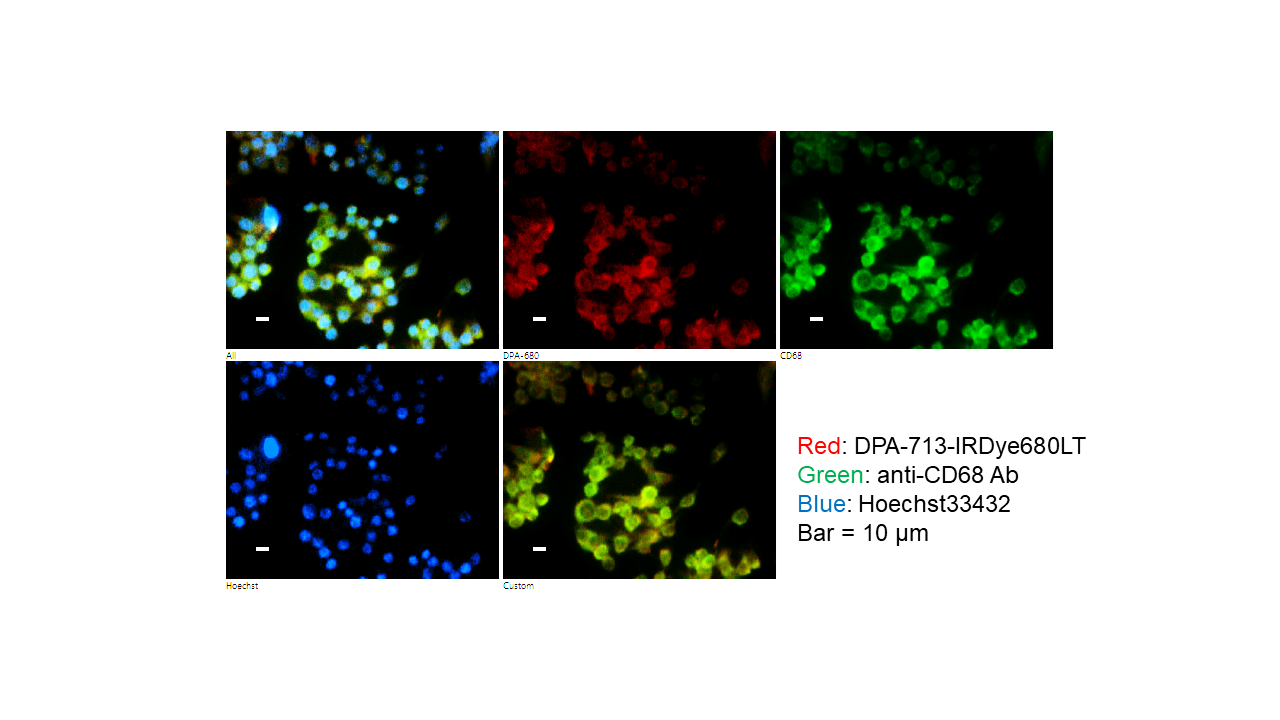

Supplement: Supplementary file 4 — Supplementary Material 4 [file 13550_2024_1157_MOESM4_ESM.tif]
