# Supplementary material for: Imaging tumor and ascites-associated macrophages in a mouse model of metastatic ovarian cancer
Source: EJNMMI Res. 2024 Nov 29;14:121. doi: 10.1186/s13550-024-01157-8 (PMC11607259; doi:10.1186/s13550-024-01157-8)
Supplement: Supplementary file 5 — Supplementary Material 5 [file 13550_2024_1157_MOESM5_ESM.docx]

Figure SI 1: Near IR autofluorescence ex vivo imaging of ovaries and uterus in healthy mice across the estrus cycle. Beginning from left to right, the uterus in proestrus observes a high degree of 700&#x00A0;nm emission autofluorescence in healthy mice, corresponding with thickening of the uterine lining and increasing levels of estrogen. In estrus, metestrus and diestrus, the observed autofluorescent signal reverts to a low background in the uterus while the ovaries remain optically inert.
